# Awareness of the Risk of Exposure to Infectious Material and the Behaviors of Polish Paramedics with Respect to the Hazards from Blood-Borne Pathogens—A Nationwide Study

**DOI:** 10.3390/ijerph14080843

**Published:** 2017-07-27

**Authors:** Anna Garus-Pakowska, Mariusz Górajski, Franciszek Szatko

**Affiliations:** 1 Department of Hygiene and Health Promotion, Medical University of Lodz, 90-647 Lodz, Poland; franciszek.szatko@umed.lodz.pl; 2 Department of Econometrics, University of Lodz, 90-214 Lodz, Poland; mariuszg@math.uni.lodz.pl

**Keywords:** behaviours, infectious material, knowledge, occupational exposure, paramedics

## Abstract

(1) Background: To determine paramedics’ frequency of contact with blood and other body fluids, as well as the analysis of knowledge of paramedics about blood-borne infections, their attitudes to patients infected with blood-borne viruses, and the post-exposure procedures implemented by paramedics; (2) Methods: An anonymous questionnaire among 190 paramedics working in various health care facilities in Poland (adjusted response rate, 76.3%); (3) Results: 78% of paramedics had contact with potentially infectious material at least several times a week. Paramedics’ knowledge on transferring infection was insufficient. Paramedics with longer employment time and better professional experience suffered fewer injuries with used needles/medical tools (*p* = 0.079). Most frequently reported factors that prevented the use of personal protective equipment were emergency situations (19.5%), skin irritations and contact allergies (19%) and, in the case of protective gloves, reduced manual dexterity (16%). In total, 82% of paramedics were concerned about the risk of being infected with HIV, HBV or HCV as a result of performing their job. In total, 97% of paramedics behaved more carefully while caring for infected patients. In total, 90% of the paramedics never refrained from performing the specific procedures necessary to help the patient whom they knew to be infected; (4) Conclusions: Despite the paramedics’ insufficient theoretical knowledge about the risk of blood-borne infections, the emphasis in the training of future paramedics should be on classes perfecting practical skills, because growing experience significantly reduces the risk of injury.

## 1. Introduction

Because of the nature of their job, paramedics in particular, are exposed to a number of factors that may have a direct or indirect impact on the risk of an accident at work.

It is the professional responsibility of the rescuer to provide quick and efficient assistance. He/She is called in emergency and life-threatening conditions, and he goes to accidents and catastrophes. The rescuer’s task is to preserve, restore and maintain basic vital functions. Medical rescuers often work under the pressure of time, in stressful situations, take responsibility for the lives of patients, often alone in the face of danger [1]. The multitude of risk factors, and in particular exposure to extreme stressful events, make paramedics, alongside firefighters, vulnerable to post-traumatic stress disorder [2]. Risk factors in the work of medical rescuers include accidents at work, including ambulance accidents [3], but also droplet-related infections [4] and sharp injuries. In the case of percutaneous exposure, assessment of the occupational risk depends on the type of performed procedures which are, or are not likely, to involve the risk of a needlestick injury. It is important to determine whether the paramedic has regular contact with blood or other potentially infectious body fluids, whether he/she is at risk of needlestick injury (i.e., injury with needle or other contaminated sharp instrument), and whether the infection can be transmitted through the injured skin or mucous membranes, and what the frequency is of these risky situations. Leiss et al. showed that over 20% of U.S. paramedics are exposed to blood each year [5]. The risk of infection of the exposed person (from a single needlestick injury by a contaminated needle) is estimated to range between 10–30% for HBV [6] and 1.8–10% for HCV [7]. It is much higher than the risk of HIV infection, which for a single contact percutaneous puncture is assumed to be 0.3%, and for the exposure through the mucous membrane the corresponding number is 0.1% [4]. In 2000, worldwide, contaminated syringe needles caused 21.7 million cases of HBV, 2 million of HCV, and 260,000 cases of HIV infection [8,9]. Thus, considering that medical workers are exposed to infectious material, each healthcare facility should run a register of possible occupational exposures, and every hospital worker (not just medical) should know the obligatory procedures to be implemented after contact with potentially infectious material. Each case of such exposure, whether it happened to the vaccinated or non-vaccinated staff, should be reported to the doctor (or the Infection Control team) responsible for the implementation of the post-exposure procedure, whose duty is to assess the likelihood of infection and instigate appropriate post-exposure procedure, depending on the type of the involved exposure [10]. Unfortunately, it is estimated that many of these events are not documented in any manner (i.e., not notified to superiors or responsible persons) [11,12]. Knowledge of the risk of infection, routes of transmission, and possible prevention is an important aspect in the development of appropriate behavior of paramedics in response to exposure to infectious material. The knowledge of the prevalence of risky events and barriers in their reporting will be essential for preventive medical care decision-makers.

The aim of the study was to determine the frequency of contacts of the paramedics with blood and other body fluids, as well as the analysis of knowledge of paramedics about blood-borne infections, their attitudes to patients infected with blood-borne viruses, and the post-exposure procedures implemented by the paramedics.

## 2. Materials and Methods

The present work is a study on exposure to infectious material of healthcare workers in Poland. The study included 190 selected paramedics (medical rescue workers) employed in various health care facilities in Poland.

### 2.1. Sample Selection and Study Protocol

Study participants (respondents) were chosen in two ways. Firstly, we randomly selected several hospitals in Poland, to which we sent the study design, including a survey for rescuers. Secondly, we chose all working students (as rescuers) of Public Health, Medical University of Lodz. During the planning of the study, in Poland, only one higher education institution was offering paramedics Masters complementary. This ensured a cross-cutting study because the students came from different parts of the country, and they were economically active. Research was conducted in the academic year 2013/2014 and 2014/2015.

We created a questionnaire after a thorough review of literature (Supplementary Materials). The reliability and validity of the survey have been assessed on the basis of our own experience and previous studies conducted in our department. Anonymous questionnaire sheets comprising 32 questions were distributed among the paramedics. The questions concerned the frequency and type of contact with infectious material, the use of personal protective equipment, attitudes to patients infected with blood-borne viruses, as well as the knowledge on such infections. Duly completed questionnaire sheets were obtained from 145 emergency medical technicians (paramedics); the adjusted response rate was 76.3%.

In the present analysis, the following sociodemographic measures were taken into account: gender, age, work experience (in years), number of work posts. Educational level was categorized as Master of Public Health—Emergency Medicine Expert, Licensed paramedic and registered paramedic.

### 2.2. Statistical Analysis

We used nonparametric tests for independence and correlation in order to study the existence of stochastic relationship between variables describing the general population. We decided to use three tests: Pearson’s chi-squared test and its modification for nominal variables based on V-Cramer statistics, and Spearman test, which also indicated the trend of that relationship. Besides, to compare several of the dependent groups, we calculated the Kendall’s coefficient of concordance. The statistical computations were performed using IBM SPSS Statistics 20 (Chicago, IL, USA) and Excel. The level of statistical significance was set at *p* ≤ 0.05.

The study protocol was approved by the Bioethics Committee of the Medical University of Lodz (Document No. RNN /163/14/KB of 11.02.2014) in full accordance with the Declaration of Helsinki of the World Medical Association. Informed written consent was obtained from each participant. Data were made anonymous before analysis.

## 3. Results

### 3.1. Characteristics of the Study Group

Most of the 145 Paramedics Studied were young men not older than 29 years of age, with higher education, with a short work experience, employed at one or two posts, Table 1.

### 3.2. The Frequency of Contact of the Paramedics with Blood and Other Body Fluids

The paramedics had constant contact with potentially infectious material. In our study, 78% of the paramedics reported that they had such contact at least several times a week, including 41.4% of paramedics experiencing such contact about a dozen times a day.

The most common response to the question about exposure to infectious material in the last 12 months preceding the survey was “several times a year through intact skin” (41% participants). Of those paramedics who experienced the exposure (within the last year) to infectious material through the damaged skin, mucous membranes or by splattering onto the conjunctiva of the eye, the answer “several times a year” was also the most common (25% of the paramedics had contact through damaged skin, 30.5% women vs. 20.9% men; 15% through the mucous membranes, 18.6% women vs. 12.8% men and 17% through the conjunctiva of the eye, 16.9% women vs. 16.3% men). Statistically significant differences were noted only for the contact with potentially infectious material through the intact skin; men were significantly more likely to experience such exposure. The distribution of contacts with infectious materials is presented in Table 2.

Obvious exposures include needlestick punctures with used needles or other sharp contaminated instruments. During their total working lives, 39% of the paramedics experienced such risk of infection. The Spearman test showed that people with longer employment time and better professional experience suffered fewer injuries with used needles/medical tools (during blood collection, injections, but also after the cleaning of tools). This relationship was at the limit of statistical significance (*p* = 0.079). The number of injuries did not depend on the number of work posts held by the paramedic.

We also asked about the handling of used needles. Most of the paramedics (65%) placed the needle in a special container, immediately after use. However, as much as one-third of the study subjects reported that they usually proceeded correctly, but occasionally they used to replace the protective cap on the needle, thus contributing to an increased risk of injury. Paramedics with higher education more often replaced the cap on the needle before placing in the container (Chi^2^ = 14.3; *p* = 0.006). Although all health care facilities were provided with special containers for infectious waste, 8% of study subjects claimed that there were far too few of such containers. At the same time, 11% of study participants reported that the containers were available but were not properly labeled. Please note that up to 52% of the paramedics said they sometimes made mistakes and tossed infectious material into a wrong container. Among those who admitted to doing so, the vast majority (75%) made a mistake because of the rush. No one reported placing the used needle in a wrong container because the latter was incorrectly marked.

### 3.3. The Applied Methods of Prevention

Gloves were the most common personal protective means employed by the paramedics. They were used always by 90% of the subjects. In addition, 51% of study participants reported that sometimes (when appropriate) they wore two pairs of protective gloves. As much as 38% of the paramedics did not use visors, such as a mask or goggles.

Most frequently reported factors that prevented the use of personal protective means were emergency situations (19.5%), skin irritations and contact allergies, e.g., to latex (19%) and, in the case of protective gloves, reduced manual dexterity (e.g., impaired ability to palpate veins) (16%). Every tenth paramedic (12%) also suggested that training on occupational health and safety was not sufficient and therefore he/she did not know when the use of protective gloves was advisable, and when it was not required.

Insufficient knowledge about the ways of transmitting infections, and the need for protective measures, may also contribute to the failure to use the visor shields. As many as 55% of respondents said that, although they generally used protective gloves, sometimes they would take them off to make it easier to serve the patient. Those behaviors were affected by work experience, level of education and the number of work posts. Paramedics with longer years of service, more frequently reported that they removed gloves during work (Chi^2^, *p* = 0.031; Spearman test, *p* = 0.007). However, increased education levels tended to decrease the frequency of removing protective gloves (Spearman, *p* = 0.024, Kendall’s test, *p* = 0.023). Paramedics employed at several work posts rarely took off their gloves (Chi^2^, *p* = 0.003; Spearman, *p* = 0.003). We observed little correlation between the opinion on the effectiveness of preventive measures and the frequency of removing the gloves. Paramedics who believed that hygiene measures do not protect employees from infection often took off the protective gloves when working with a patient (Chi^2^ = 8.793, *p* = 0.062).

Subjective self-assessment of compliance with hygiene procedures looked to be very good (49% of study subjects confirmed that they had always followed the recommended procedures, and 48% that they occasionally neglected the procedures, but the instances of negligence were rare). However, assessment of compliance with hygienic procedures by co-workers was more restrictive. Only less than 19% of the paramedics claimed that their colleagues’ compliance with the safety rules was very good, and the majority (63%) said that their colleagues’ compliance with the rules was only satisfactory. Every tenth paramedic (12%) rated colleagues’ compliance as very poor, and 7% of the participants said they did not pay attention to it. The men evaluated their colleague’s behaviors as poor more often than women (Chi^2^; *p* = 0.071).

### 3.4. Knowledge of Blood-Borne Infections and Their Prevention

A short knowledge test consisted of five questions where the possible answers were “true”, “false” or “do not know”. The paramedics by far least frequently (33.8%) answered correctly to the question, “Is tuberculosis communicable only by droplet contact?” Other questions also revealed insufficient knowledge of the study participants. For example, only 59% of the paramedics knew that up to 60% of infections with hepatitis B in Poland are nosocomial, and every fifth participant (17.9%) claimed that in the case of a single puncture with a contaminated needle, one is more likely to become infected with HIV than HBV. The value of the average number of correct answers given by the paramedics was 3.5, and the differences in the responses did not depend on education (Chi^2^ = 6.882; *p* = 0.549), gender (Chi^2^ = 2.94; *p* = 0.568) or years of employment (Chi^2^ = 15.152, *p* = 0233). The Spearman test showed a significant correlation (*p* = 0.058) between education and the knowledge, and please note that the answers of the paramedics with higher education were less frequently correct than those of their colleagues. Table 3 shows the distribution of the answers in the test of knowledge.

Unfortunately, most of the answers of the paramedics (46.75%) were based on the knowledge gained at school/college, and only every fifth paramedic (18.3%) received extra information during various types of supplementary courses and an equal number of responders (18.3%) expanded their knowledge by reading recent scientific journals. Our study indicated that training courses on post-exposure procedures were organized in workplaces too rarely (30%), or even were not run at all (27%). Every fifth paramedic (21%) had not heard of such training.

### 3.5. Attitudes of the Paramedics toward Patients Infected with Blood-Borne Viruses and Procedures Implemented after Occupational Exposure to Infectious Materials

As much as 82% of paramedics were concerned about the risk being infected with HIV, HBV or HCV as a result of performing their job. At the same time, 37% of study subjects knew in person a paramedic who had been infected as a result of the occupational exposure. Persons with higher education (Chi^2^, *p* = 0.03; Kendall, *p* = 0.01) and males (Chi^2^, *p* = 0.027) reported more frequently that they knew people who had been infected at work.

Changing their behavior under the influence of concern about their own health is a major problem among people dealing with patients on a daily basis. Almost all of the paramedics (97%) confirmed that the necessity to help people they knew to be infected with HIV, HBV or HCV resulted in them behaving more cautiously in dealing with these patients. The change of the behavior was not affected by any of the analyzed variables.

At the same time, almost all (90%) of the paramedics never refrained from performing specific procedures necessary to help the patient whom they knew to be infected. Seven per cent of the paramedics confessed that they had abandoned the care of an infected patient as a result of the concern for their own health. Study participants with higher education were less likely to refrain from serving the infected patient (Chi^2^, *p* = 0.208). However, male paramedics more frequently admitted that they had not provided help to the infected patient and that they refused to help the patient (Chi^2^, *p* = 0.044).

The concern of paramedics about their own safety did not go hand in hand with obeying the duty to report all incidents of exposure to infectious material. Although 85% of the paramedics confirmed their obeying the duty to report incidents of exposure to infectious material, 15% of the study subjects did do not do that—either failing to perceive their failure to report as a threat to themselves, or claiming that these reports “are of no consequence”. The paramedics with the shortest and the longest experience, as well as those with higher education, more often reported cuts/punctures by sharp tools to their superiors (Chi^2^, *p* = 0.1; Kendall, *p* = 0.06). This trend is confirmed by the responses to the question “Do you think that the implemented hygienic measures and vaccination protect workers from possible risks associated with their work?” As many as 41% of the paramedics were of the opinion that the practical value of the hygienic measures and post-exposure procedures intended to protect workers from the risk of occupational infection is doubtful.

## 4. Discussion

Exposure of medical personnel to infectious material is an important issue for the implementation of safe tools and safe containers for used tools. Of particular interest are the factors responsible for such injuries. The knowledge of those factors is essential for the development of methods to eliminate them. In our study, we have shown that medical rescuers have frequent contact with potentially infectious material and that a large proportion of paramedics have already suffered injuries in their previous careers. That is a lot, when we remember that the majority of the study subjects were young, with short work experience (<15 years). The frequency of injuries decreased significantly with increasing seniority and growing professional experience. The number of work posts (and thus the general workload) did not affect the risk of injury. In the USA, exposure to infectious material during the year preceding the survey was reported by 15% of the paramedics [13].

Replacing the cover back onto the needle increases the risk of injury [14]. Such behavior was reported more frequently by paramedics with higher education. However, it should be noted that in Poland there has been a change in the system of paramedics training (now the professional title of a paramedic is obtained after completion of a 3-year course). Therefore, paramedics with higher education are primarily all a bunch of young people with short work experience—less experienced, more likely to commit mistakes.

The use of personal protective equipment reduces the risk of the occupation-related infection. Gloves were the most commonly used protective means. The subject’s opinion on whether the gloves are truly able to protect the wearer from infection constitutes an interesting aspect of the problem: paramedics who believed that hygiene measures do not protect employees from infection more often used to take off the gloves to “facilitate” manual execution of tasks while working with the patient.

Peer assessment of compliance with hygiene procedures can be a valuable cognitive tool. It makes it possible to estimate the degree of reliability of the reported self-evaluation. Most of the paramedics rated their own adherence to hygienic procedure highly. However, worse ratings were given for their colleagues. Confrontation of self-assessment results with those from the assessment offered by co-workers may indicate that the results of various studies using questionnaire methods may be considerably biased and do not reflect the actual degree of compliance with recommended hygiene procedures [15,16,17].

Our study confirmed earlier that the barriers to the proper use of personal protective equipment include emergency situations and allergies. One in ten participants admitted that more training on that subject would be advisable. In a study by Mathews et al., the opinion was shared by each fifth paramedic [18].

The level of knowledge about blood-borne infections and the risk of infection among the examined paramedics was rather insufficient. This can be explained by an insufficient number of training courses organized by companies and the fact that the moiety of the paramedics’ knowledge was based on the information obtained during basic training. Payments received by paramedics in Poland are not high. The average paramedics’ salary does not exceed the national average salary (about 3900PLN/$1030 in 2015), and such remuneration is often achieved by hiring in several workplaces. This, in turn, results in fatigue and lack of time for self-training. In addition, the costs of most commercial training courses often exceed the financial capacity of young paramedics. Interestingly, in the test of knowledge, correct answers were more frequent among paramedics with lower education. However, you have to remember that those were mainly people with greater seniority, and therefore with more practical experience. For organizers of training courses for paramedics, this should be a signal that the education of this professional group should include a large proportion of practical classes.

The vast majority of the paramedics were afraid of being infected during work, and almost all admitted that they performed medical procedures more carefully with patients they knew to be infected with HIV, HBV or HCV. This may indicate a lack of awareness that absolutely every patient should be treated as a potential source of infection. The use of personal protective equipment for operations performed on the patient and carefully carrying out all procedures should not be dependent on the results of the patient’s tests for markers of blood-borne virus infections, due to the possibility of patients being in the serological window or low sensitivity of the tests. The patient him/herself is not obliged to inform the provider of the service that he/she is infected, hence during a procedure involving possible contact with potentially infectious material, the same safety precautions should be used for all patients. Only Arenas et al. reported that hygiene-related behaviors were not affected by the awareness of the medical personnel that the patient was infected [19]. The mission of paramedics is to help and save lives. Fortunately, most paramedics did not refuse to provide such assistance to patients known to be infected with blood-borne viruses. On the other hand, the concern for their own health should entail the need to report an incident of exposure to infectious material, and then to implement the post-exposure procedures. However, 15% of the paramedics did not report to their superiors that the exposure had occurred, most often—as in the study by Sohn et al. [20]—because of their conviction that the risk of infection was low. Almost half (41%) of study subjects thought that the procedure carried out post-exposure did not reduce the risk of infection. This again suggests the need for continuing education among health care workers.

## 5. Conclusions

Paramedics’ knowledge about blood-borne infections and their prevention is insufficient, and this implies a negative patterns of behavior towards infected patients. Despite the insufficient theoretical paramedics’ knowledge about the risk of blood-borne infections, the emphasis in the training of future paramedics should be on classes perfecting practical skills, because growing experience significantly reduces the risk of injury.

### Limitations

The main disadvantage of the presented study is its tool—a questionnaire—which can itself be misleading. As we highlighted in the Discussion, the responses received were subjective and there is always the risk of overestimating the results in “sensitive” questions such as behavior or opinion of respondents.

## Figures and Tables

**Table 1:** Characteristics of the study group.

Characteristics of the Study Group	No. of Paramedics	Percentage of Paramedics (%)
**Gender**		
Women	59	40.7
Men	86	59.3
Total	145	100.0
**Education**		
Master of Public Health—Emergency Medicine Expert	91	62.8
Licensed paramedic	11	7.6
Registered paramedic	43	29.7
Total	145	100.0
**Age (years)**		
20–29	113	77.9
30–39	18	12.4
40–49	10	6.9
>50	2	1.4
Total	145	100.0
**Work experience (years)**		
<5	95	65.5
5–15	5	24.1
16–25	40	6.9
>25	5	3.4
Total	145	100.0
**Number of work posts**		
1	74	51.0
2	53	36.6
3 or more	18	12.4
Total	145	100.0

**Table 2:** Frequency of paramedics’ contacts with potentially infectious material within 12 months preceding the study.

Exposure Type	Gender	Frequency of Exposure within the Last 12 Months											
		Everyday		10 to 19 Times		1 to 9 Times		Once		Never		Statistical Significance	
		N	%	N	%	N	%	N	%	N	%	Chi^2^	*p*-Value
Through intact skin	f	2	3.4	4	6.8	24	40.7	6	10.2	23	39.0	7.983	0.092
	m	0	0.0	9	10.5	35	40.7	19	22.1	23	26.7		
Through damaged skin	f	0	0.0	2	3.4	18	30.5	10	16.9	29	49.2	2.312	0.51
	m	0	0.0	5	5.8	18	20.9	13	15.1	50	58.1		
Through mucous membranes	f	1	1.7	3	5.1	11	18.6	6	10.2	38	64.4	2.609	0.625
	m	0	0.0	6	7.0	11	12.8	10	11.6	59	68.6		
Splattering onto conjunctiva	f	0	0.0	0	0.0	10	16.9	9	15.3	40	67.8	1.539	0.673
	m	0	0.0	2	2.3	14	16.3	11	20.8	59	68.6		

Note: f—female, m—male; Chi^2^—Chi—square test of independence.

**Table 3:** Distribution of the responses provided by the paramedics during the test of knowledge.

Question	Correct Answer		Incorrect Answer		No Answer		Total	
	N	%	N	%	N	%	N	%
Can use of gloves replace disinfection of hands?	134	92.4	10	6.9	1	0.7	145	100
Is disinfection of hands necessary in emergency situations?	107	73.8	37	25.5	1	0.7	145	100
Do you agree that 60% of HBV infections in Poland is connected with health care?	86	59.3	56	38.6	3	2.1	145	100
Is it more likely to become infected with HIV than with HBV as a result of single needlestick injury with a contaminated needle?	117	80.7	26	17.9	2	1.4	145	100
Is infection with tubercule bacillus possible solely through droplet infection?	49	33.8	93	64.1	3	2.1	145	100

## Supplementary Materials

The following are available online at www.mdpi.com/1660-4601/14/8/843/s1, The Questionnaire.
