# Supplementary material for: Awareness of the Risk of Exposure to Infectious Material and the Behaviors of Polish Paramedics with Respect to the Hazards from Blood-Borne Pathogens—A Nationwide Study
Source: Int J Environ Res Public Health. 2017 Jul 27;14(8):843. doi: 10.3390/ijerph14080843 (PMC5580547; doi:10.3390/ijerph14080843)
Supplement: Supplementary file 1 [file ijerph-14-00843-s001.pdf]

Dear Sir or Madam,

In the Department of Hygiene and Epidemiology, Medical University of Lodz, we conduct a study on the exposure of workers to medical infectious material. We believe that given by Mr / Ms answers will contribute to greater interest in occupational health and safety, so that every employee feel comfortable and safe. The information gathered will also help to identify risky behavior committed consciously or unconsciously by the medical staff.
Please Mr / Ms to provide honest answers. We ensure that the survey is completely anonymous. The information collected will be developed in the form of aggregated statistics.
***Thank you for participating in the study*.**

QUESTIONNAIRE

1. Whether in the course of your work, there were situations risky, threatening the safety of you? (during the last two years)

a. yes, very often 1a ☐

b. yes, from time to time 1b ☐

c. very rarely 1c ☐

d. no, never 1d ☐

2. What kind of situations they were?

…………………………………………………………………………………………………
…………………………………………………………………………………………………
…………………………………………………………………………………………………

3. How often has you in contact with patient's blood, body fluids, secretions or excretions?

a. few / several times a day 3a ☐

b. a few times a week 3b ☐

c. several times a month 3c ☐

d. several times a year 3d ☐

e. never 3e ☐

4. How often have you used personal protective equipment?

	always	often	sometimes	never
protective gloves				
double pair of protective gloves				
protective mask				
protective goggles				
protective clothing				

5. What according to you affects the use of protective gloves?
(Please select no more than 3 main reasons for non-use of protective gloves)

a. lack of knowledge about when to use gloves 5a ☐

b. reducing manual dexterity, worse sensing wires, etc. 5b ☐

c. contact allergies to latex, skin irritation 5c ☐

d. the hospital does not have cosmetics protecting skin 5d ☐

e. lack of time for the establishment of the gloves, too heavy workload 5e ☐

f. emergency situations 5f ☐

g. frequent shortages of gloves 5g ☐

h. lack of proper size gloves 5h ☐

i. restrictions "of management" for excessive wear gloves 5i ☐

j. there is no infections associated with not using gloves (on the hospital unit) 5j ☐

k. others ....

6. Have you ever removed the protective gloves to "easier" perform the operation with the patient?

a. yes, very often 6a ☐

b. yes, but occasionally 6b ☐

c. no, never 6c ☐

d. I do not use the protective gloves 6d ☐

7. How do you assess yours compliance with the procedures of hygiene (hand washing, donning gloves, etc)?

a. always I comply with the procedures 7a ☐

b. sometimes I do not comply with the procedures 7b ☐

c. quite often I do not comply with the procedures 7c ☐

d. very often I do not comply with the procedures 7d ☐

e. in the ward where I work, there is no specific, implemented procedures 7e ☐

8. How do you assess the compliance of hygienic procedures by your colleagues?

a. very good/good 8a ☐

b. acceptably 8b ☐

c. bad, very bad 8c ☐

d. I do not care about this 8d ☐

9. When you performed vaccination against hepatitis B?

a. a year ago 9a ☐

b. 2 years ago 9b ☐

c. 2-5 years ago 9c ☐

d. more than 5 years ago, but I controlled the level of anti-HBS 9d ☐

e. more than 5 years ago, and I did not control the level of anti-HBS 9e ☐

f. I was not vaccinated 9f ☐

g. I do not remember whether I was vaccinated 9g ☐

10. Do you feel you performed oral hygiene and vaccination protect workers from possible risks associated with the work?

a. yes 10a ☐

b. no 10b ☐

c. I do not know 10c ☐

11. Please indicate how often you have had contact with potentially infectious material (within the last year):

	never	once	several times	dozen times	every day
through intact skin					
through non-intact skin					
transmucosally					
by splattering on the mucous membranes of the eye					

12. How often you hurt the used needle or worn medical tool?

a. every day 12a ☐

b. a few times a week 12b ☐

c. several times a month 12c ☐

d. several times a year 12d ☐

e. less than a few times a year 12e ☐

f. I have never hurt 12f ☐

13. Does your workplace have special containers in which to put sharp instruments?

a. yes, they are always available 13a ☐

b. yes, they are available, but there are not enough 13b ☐

c. there is no such containers 13c ☐

14. What are you doing with a used needle? Are you throwing it in a special container or assumes you re-cap?

a. always - immediately after use - I put into the container 14a ☐

b. usually I put into a container, but sometimes I assume cap 14b ☐

c. always – at first – I assume cap 14c ☐

15. Are there (at your workplace) different containers / bags for infectious waste and non-infectious??

a. yes, they are always available and properly marked 15a ☐

b. yes, they are available but unmarked 15b ☐

c. no such containers 15c ☐

16. Have you ever thrown infectious material, along with other waste to an unmarked container / bag?

a. yes, few / several times a day 16a ☐

b. yes, several times a week 16b ☐

c. yes, several times a month 16c ☐

d. yes, several times a year 16d ☐

e. never 16e ☐

17. If you answered question 16 "yes", please write why?

a. due to the rush 17a ☐

b. lack of containers 17b ☐

c. carelessness 17c ☐

d. other reasons ....

18. Do you - after cutting, stick injury,- report this to your supervisor or the person responsible for the conduct post-exposure?

a. yes, immediately 18a ☐

b. yes, but only after some time 18b ☐

c. no, I do not see any threats 18c ☐

d. not because there is no result from the reports 18d ☐

19. Do you know a paramedic who has been infected as a result of occupational exposure?

a. yes 19a ☐

b. no 19b ☐

c. only I've heard of such a person 19c ☐

20. Are you afraid of infection?

a. yes 20a ☐

b. no 20b ☐

c. I do not know 20c ☐

21. Do you keep more careful in dealing with the patient, which is known to be infected with HIV, HBV or HCV?

a. yes 21a ☐

b. no 21b ☐

c. I do not remember 21c ☐

22. Have you ever failed to perform actions with the infected patient because you were afraid of infection??

a. yes, it happened several times 22a ☐

b. yes, but only a few times 22b ☐

c. no, never 22c ☐

23. Please respond to each statement:

	TRUE	FALSE
Can use of gloves replace disinfection of hands?		
Is disinfection of hands necessary in emergency situations?		
Do you agree that 60% of HBV infections in Poland is connected with health care?		
Is it more likely to become infected with HIV than with HBV as a result of single needlestick injury with a contaminated needle?		
Is infection with tubercule bacillus possible solely through droplet infection?		

24. Do you feel the need to increase your knowledge about the possibility of infection with?

a. yes 24a ☐

b. no 24b ☐

c. I do not know 24c ☐

25. Where do you get knowledge about the prevention of infectious diseases? *(You can select multiple answers)*

a. knowledge gained during the school / college 25a ☐

b. knowledge of scientific journals 25b ☐

c. knowledge of media (television, radio, internet) 25c ☐

d. knowledge gained at the training courses 25d ☐

e. other (please specify the source)…………………………………………………………….

26. Does your employer provide training on procedures for workers after exposure to the infection ?

a. yes, very often 26a ☐

b. yes, but too rarely 26b ☐

c. there is no such training 26c ☐

d. I do not know 26d ☐

27. When did you participate in the training on proceedings workers after exposure to the infection?

a. last month 27a ☐

b. in the last 6 months 27b ☐

c. in the last year 27c ☐

d. more than year ago 27d ☐

e. over 5 years ago 27e ☐

f. I did not participate in such training 27f ☐

Please also Mr / Ms to write some information about yourself:

28. Gender: F ☐ M ☐

29. Age (please give a finite number of years): ....

30. Education:

a. registered paramedic 30a ☐

b. licensed paramedic 30b ☐

c. Master of Public Health – Emergency Medicine Expert 30c ☐

31. Number of work posts:

a. 1 31a ☐

b. 2 31b ☐

c. 3 or more 31c ☐

32. Work experience (years):

a. < 5 years 32a ☐

b. 6-15 years 32b ☐

c.16-25 years 32c ☐

d. > 25 years 32d ☐

Thank you for filling in the questionnaire.
